# Supplementary material for: Complexome profiling of the Chlamydomonas psb28 mutant reveals TEF5 as an early PSII assembly factor
Source: Plant Cell. 2025 Mar 17;37(6):koaf055. doi: 10.1093/plcell/koaf055 (PMC12164590; doi:10.1093/plcell/koaf055)
Supplement: koaf055_Supplementary_Data [file koaf055_supplementary_data.zip › psb28_interactive.zip!/plots/intensity/qualityPlots/peptides/TAB2_Cre17.g702500.t1.2.html]

### Cre17.g702500.t1.2

Trivial name: TAB2  
SeqCoverage [median]: 6.10  
Mass: 39.86 kDa  
  
 

| Sequence | MissedCleavage | Mass [Da] | wtIntensity | muIntensity | Score |
| --- | --- | --- | --- | --- | --- |
| ILAQAGAERPEK | 1 | 1281.70 | 796.59 | 727.70 | 59.57 |
| LESVYKQDPR | 1 | 1233.64 | 517.83 | 0.00 | 43.31 |
|  |  |  | 1314.4 | 727.7 |  |
